# Genomic analysis of *Neisseria elongata* isolate from a patient with infective endocarditis

**DOI:** 10.1002/2211-5463.13201

**Published:** 2021-06-15

**Authors:** Marcos André Schörner, Hemanoel Passarelli‐Araujo, Mara Cristina Scheffer, Fernando Hartmann Barazzetti, Jessica Motta Martins, Hanalydia de Melo Machado, Jussara Kasuko Palmeiro, Maria Luiza Bazzo

**Affiliations:** ^1^ Molecular Biology, Microbiology and Serology Laboratory Federal University of Santa Catarina Florianopolis Brazil; ^2^ Department of Biochemistry and Immunology Biological Sciences Institute Federal University of Minas Gerais Belo Horizonte Brazil; ^3^ Polydoro Ernani de São Thiago University Hospital Federal University of Santa Catarina Florianopolis Brazil; ^4^ Department of Clinical Analysis Federal University of Santa Catarina Florianopolis Brazil

**Keywords:** cgMLST, *Neisseria* sp., pangenome, resistome, virulence factor

## Abstract

*Neisseria elongata* is part of the commensal microbiota of the oropharynx. Although it is not considered pathogenic to humans, *N. elongata* has been implicated in several cases of infective endocarditis (IE). Here, we report a case of IE caused by *N. elongata* subsp. *nitroreducens* (Nel_M001) and compare its genome with 17 N.* elongata* genomes available in GenBank. We also evaluated resistance and virulence profiles with Comprehensive Antibiotic Resistance and Virulence Finder databases. The results showed a wide diversity among *N. elongata* isolates. Based on the pangenome cumulative curve, we demonstrate that *N. elongata* has an open pangenome. We found several different resistance genes, mainly associated with antibiotic efflux pumps. A wide range of virulence genes was observed, predominantly pilus formation genes. Nel_M001 was the only isolate to present two copies of some pilus genes and not present *nspA* gene. Together, our results provide insights into how this commensal microorganism can cause IE and may assist further biological investigations on nonpathogenic *Neisseria* spp. Case reporting and pangenome analyses are critical for enhancing our understanding of IE pathogenesis, as well as for alerting physicians and microbiologists to enable rapid identification and treatment to avoid unfavorable outcomes.

## Graphical abstract

We report a case of a 71‐year‐old male with infective endocarditis caused by *Neisseria elongata*. This microorganism was isolated from blood culture, and its whole genome was sequenced. We also performed a comparative genomic analysis with seventeen *N. elongata* genomes available in GenBank to explore virulence and resistance in *N. elongata*, as well as its pangenome.

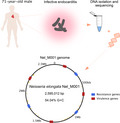

**Abbreviations**
ANI: average nucleotide identity
bpm: beat per minute
CARD: Comprehensive Antibiotic Resistance Database
cgMLST: Core Genome Multilocus Sequence Typing
IE: infective endocarditis
IU: International units
IV: Intravenous
PLSDB: plasmid database
SNPs: single nucleotide polymorphisms
VFDB: Virulence Factor Database

*Neisseria elongata* is part of the commensal microbiota of the oropharynx and is unusual among *Neisseria* spp. in that it is a rod‐shaped bacterium, whereas most of the other members of the genus are coccus‐shaped [1]. Its identification and classification as a new species were proposed in 1970 by Bovre and Holten [1]. Its possible pathogenicity was only reported in 1983, when a 31‐year‐old woman with known mitral valve prolapse and valve replacement requirement developed infective endocarditis (IE) [2].

Commensal and pathogenic *Neisseria* spp. are competent for DNA uptake by transformation, an important mechanism for horizontal genetic exchange. Accessory genes are known to be broadly shared among members of the genus [3, 4]. To date, three subspecies of *N. elongata* have been identified based on biochemical tests: subsp. *elongata* [1], subsp. *glycolytica* [5], and subsp. *nitroreducens* [6]. Biochemical classification can identify pathogenic *Neisseria* species but has not proved satisfactory in distinguishing other species. The application of phylogenetic approaches based on nucleotide sequencing has led to species reclassification within the genus [4] and, more recently, to the characterization of novel *Neisseria* species [7].

Three *N. elongata* subspecies were described in 1990: *N. elongata* subsp. *nitroreducens*, *N. elongata* subsp. *elongata*, and *N. elongata* subsp. *glycolytica*. This classification was initially proposed because specimens differed in acid production from d‐glucose, nitrate reduction, and catalase positivity [6]. Although not usually considered pathogenic to humans, these subspecies has been implicated in several cases of IE [8, 9].

Here, we report a rare case of IE caused by *N. elongata* subsp. *nitroreducens* that occurred in Brazil, in 2018, in a patient with a history of aortic and mitral valve replacement and recent dental procedure, evolving to abscess formation and death due to associated complications. Along with the case report, we also performed a comparative genomic analysis to explore virulence, resistance, and biochemical characteristics in *N. elongata* as well as its pangenome.

## Results and Discussion

### Clinical patient data

Twenty‐three cases of definite or possible IE caused by *N. elongata* had been reported up to 2016 [8, 9]. Thereafter, only one more case was reported [10]. A case reported in 2007 was also included in this study [11] (Table S1).

Here, we report the case of a 71‐year‐old male admitted to the university hospital of Federal University of Santa Catarina after intermittent fever for 8 days, general decline, inappetence, dizziness, and night sweats. The patient’s medical history comprised two valve replacements (aortic and mitral) in 2009 and implantation of a cardiac pacemaker in 2013. He reported a recent dental procedure and treatment with amoxicillin. On the admission, he presented with good general condition and was eupneic and hydrated. On the first day of hospitalization, the transthoracic echocardiography revealed the metallic prostheses in normal positions, suggestive image of vegetation on the ventricular face of the aortic prosthesis, moderate enlargement of the left atrium, mild dilation of the ascending aorta, and left ventricle with mild hypertrophy and good contractile function (Fig. S1A). Four blood culture bottles were collected (two for both aerobes and anaerobes), and intravenous (IV) administration of 3 000 000 IU penicillin was started. After 19 h of incubation (BACTEC™ FX/BD^®^, Becton, Dickinson, Franklin Lakes, NJ, USA), both the aerobic bottles were positive. Gram‐negative rods were detected in the sample; thus, penicillin was replaced with gentamicin (90 mg IV twice daily) and ceftriaxone (2 g IV once daily). During hospital stay, the asthenia improved, and no new complaints or intercurrences were observed, although nocturnal sweating persisted. After 3 days, the vegetation disappeared, and a suggestive image of an abscess at the base of the aortic prosthesis was observed (Fig. S1B). On the tenth day of hospitalization, a transesophageal echocardiogram showed an abscess (2.4 × 1 cm) in the aortic root (Fig. S1C), and surgical intervention was indicated.

After 11 days of hospitalization the patient had pain in the left hypochondrium, profuse sweating, tachypnea, and hypotension. He was responsive but lethargic, with a blood pressure of 50/30 mmHg and a heart rate of 45 bpm. The patient sustained two nonshockable cardiac arrests (19 and 4 min), with orotracheal intubation and return of spontaneous circulation at high doses of noradrenaline (120 mL·h^−1^). About 2 h after worsening of the state, the patient died of cardiogenic shock due to IE.

Out of the three subspecies reported, subsp. *nitroreducens* was the main causative agent of IE (80.8%, 21/26). Three and two cases were caused by subsp. *elongata* and *glycolytica*, respectively (Table S2). In 38.5% (10/26) of the cases described, the patients had performed a dental procedure or had had a dental infection, both considered risk factors for IE. A recent study on the oral tropism of *Neisseria* spp. using metagenomic data revealed a predominance of *N. elongata* in gingival plaque [12], indicating the site of the dental procedure as a probable focus of infection in the referred cases. No risk factors were reported in eight IE cases caused by *N. elongata* [6, 13, 14, 15, 16, 17, 18, 19].

The abscess formation was reported in only six cases, three in the myocardial region [2, 20, 21], one subvalvular [22], one in aortic root [21], and one in the brain [8]. These five patients had an initial vegetation on the mitral or aortic valve and had undergone valve replacement surgery. In the present case, it was not possible to obtain a sample of the abscess. In the two cases in which it was possible to collect material from the abscess, microbiological cultures were negative [2, 21]. According to European Society of Cardiology guidelines for IE management, the presence of abscess requires urgent surgical intervention (within a few days) [23, 24, 25]. Unfortunately, the patient suffered two cardiorespiratory arrests and died 1 day after surgical indication.

### Genomic features of *N. elongata* subsp. *nitroreducens* Nel_M001

Biochemical tests (d‐glucose, catalase, and nitrate reduction) were used to discriminate the subspecies, identifying the isolate Nel_M001 as *N. elongata* subsp. *nitroreducens*. The genome assembly of Nel_M001 isolate resulted in 26 scaffolds, comprising 2.5 Mbp and 54.04% G + C content. The genome coverage was 88.0x. N50 was 230 084 bp; L50, 4; and maximum scaffold length, 541 257 bp. According to BUSCO, the genome completeness was 96.2%. Nel_M001 genome contained 2661 genes and 2605 coding sequences. There were 2496 protein‐coding genes, 109 pseudogenes, and 1 CRISPR region. The number of RNA genes was 56, including 3 rRNAs (5S, 16S, and 23S), 49 tRNAs, and 4 noncoding RNAs. No plasmids were detected in Nel_M001 genome. The genome is deposited in NCBI under the accession number JAGJWT000000000.

### The classification of *N. elongata* and its pangenome

**Fig. 1:**
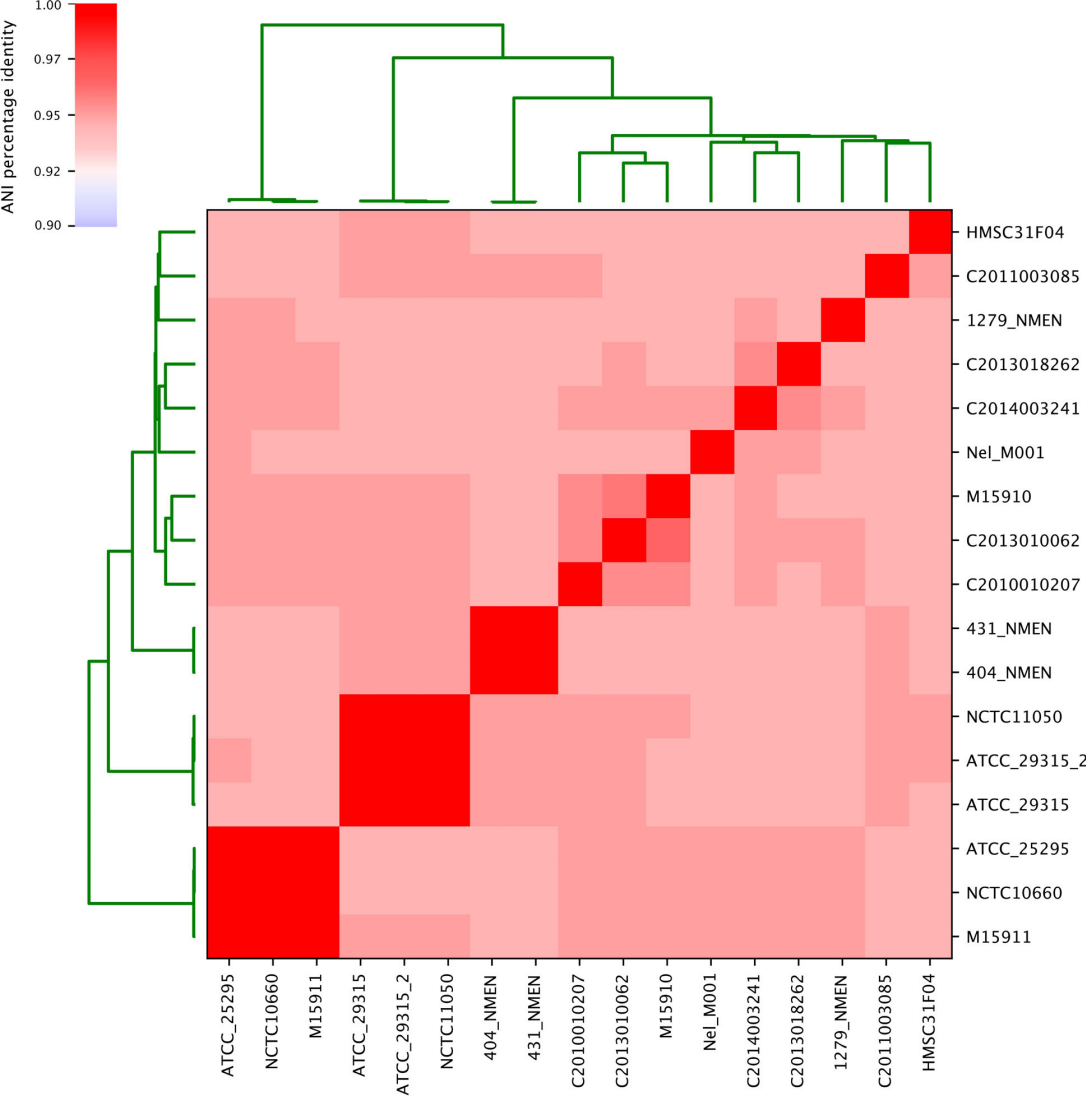
ANI among seventeen *N. elongata* genomes used in this study.

Comparative genomic analysis average nucleotide identity (ANI) provides a raw estimate to define bacterial species [16]. A minimum threshold of 95% ANI (or <0.05 mash) has been used to delineate species (Fig. 1). We obtained 2830 *Neisseria* genomes available in GenBank in August 2019 and used Nel_M001 as an anchor strain to evaluate mash distances from other *Neisseria* genomes. The ranked distribution of ANI values (1—mash) from *N. elongata* Nel_M001 showed an abrupt break around 96%, supporting the effective species delineation (Fig. S2). The presence of the type strain ATCC 25295T confirmed that this retrieved group of 17 genomes corresponds to *N. elongata*. Moreover, we also reclassified *Neisseria* sp. HMSC31F04 (GCA_001807735.1) as *N. elongata*.

**Fig. 2:**
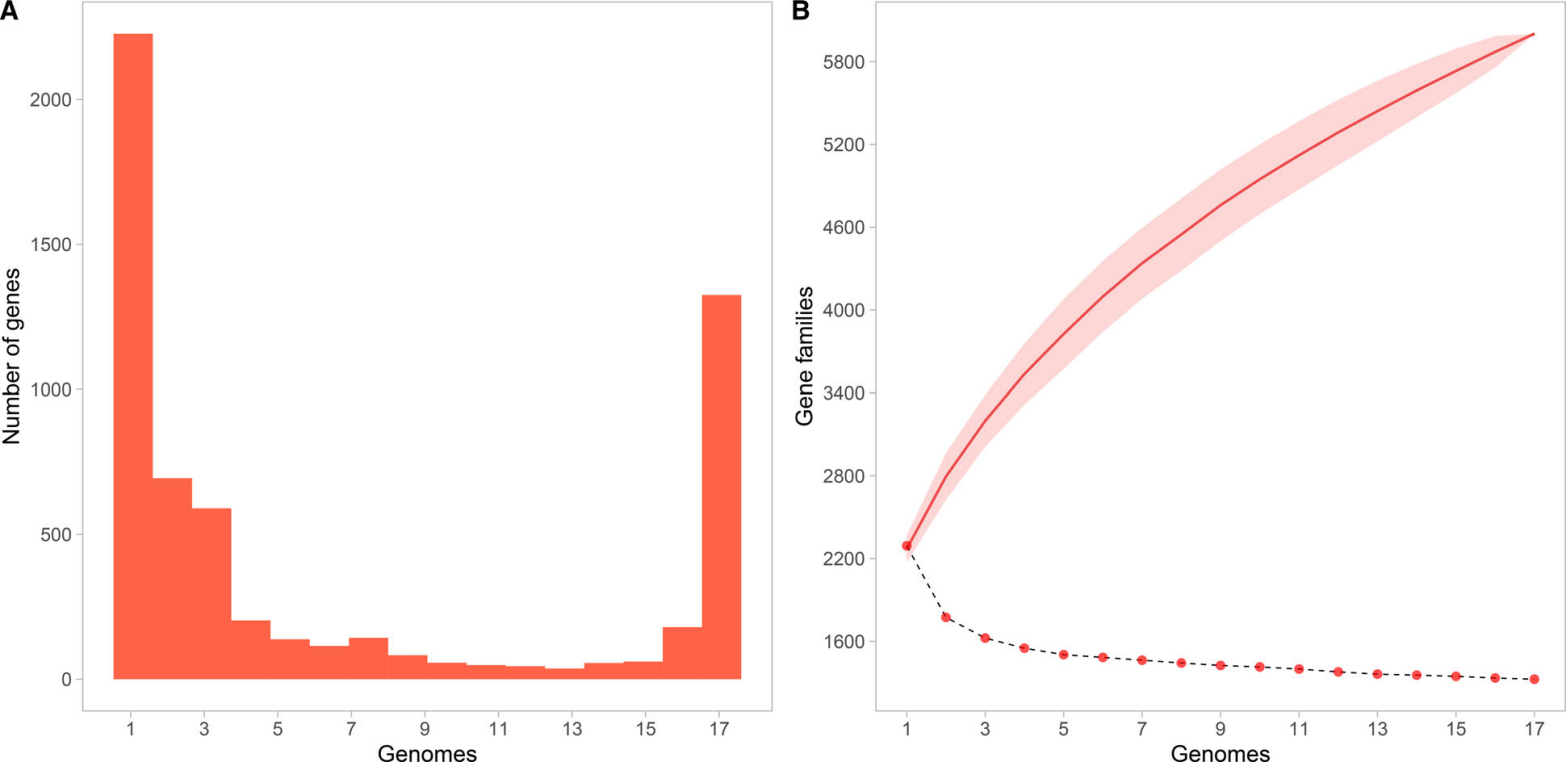
*Neisseria elongata* pangenome. (A) Distribution of the number of genes showing the highest proportion of low‐frequency genes. (B) Decay of core genome (dotted line) and the pangenome cumulative curve showing the open pangenome in *N. elongata* (red line).

After defining *N. elongata*, we estimated the pangenome, which is the total set of genes in a given species [23]. By using 17 genomes, the pangenome of *N. elongata* comprises 6003 gene families, of which 1325 constitute the core genome. The pangenome is dominated by low‐frequency genes (Fig. 2A). Moreover, Nel_M001 has the highest number of exclusive genes. Based on the pangenome cumulative curve (Fig. 2B), *N. elongata* has an open pangenome (α = 0.65). This estimated alpha is much lower than the threshold to define pangenome openness (α < 1), and it is in line with the estimated α in *N. meningitidis* and *N. gonorrhoeae*. The open pangenome in *N. elongata* implies that as we increase the number of genomes from this species to analyze, new gene families will be detected at a high rate.

**Fig. 3:**
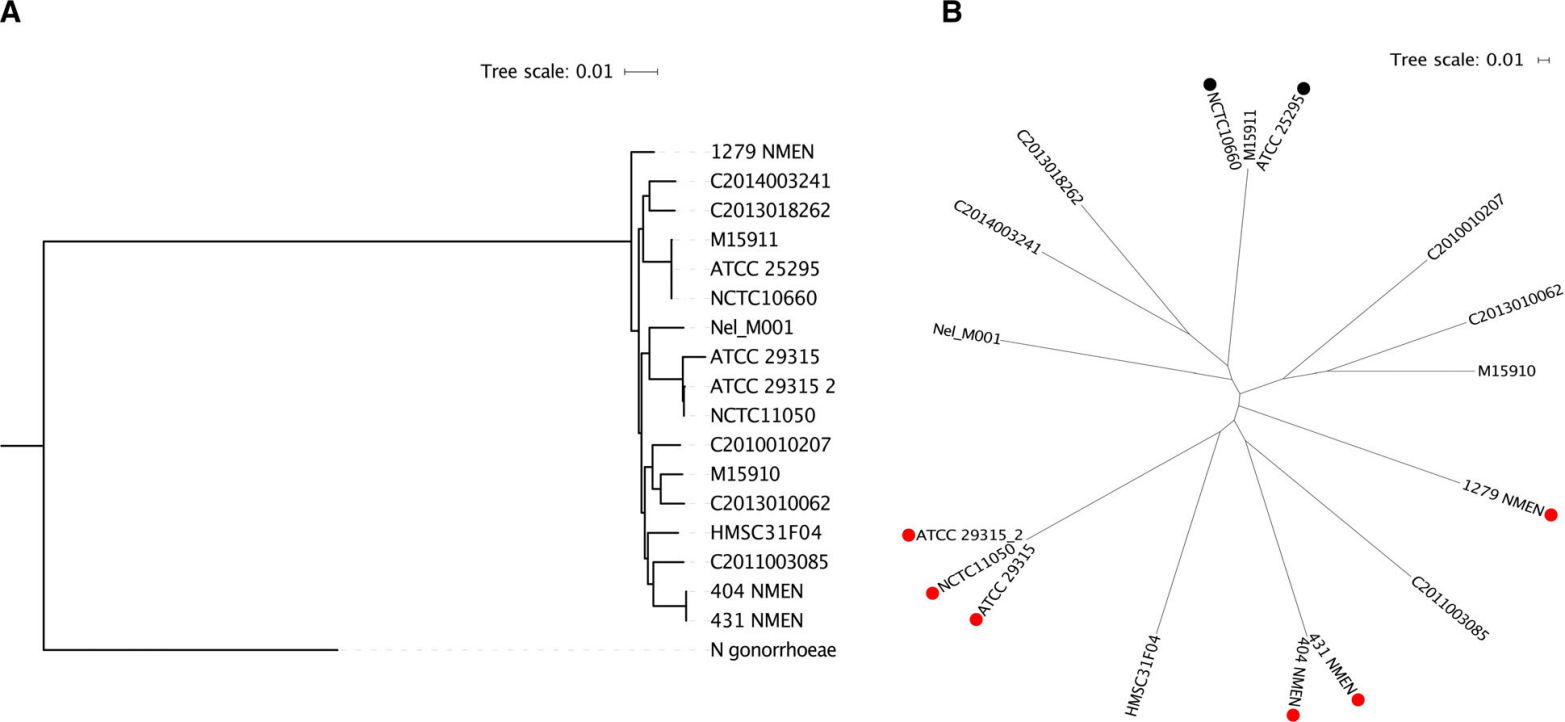
Phylogenetic tree of (A) orthologous proteins using *Neisseria gonorrhoeae* FA1090 as an outgroup and (B) SNPs extracted from core genome. Red circles and black circles represent *N. elongata* subsp. *glycolytica* and subsp. *elongata*, respectively.

Regarding the genomes included in this work, it is important to note that some isolates were sequenced and deposited more than once, because the first isolates obtained were sent to different research groups [5]. However, differences among strains of the same isolate (ATCC29315 and ATCC29315_2) were observed, as shown in the phylogenetic tree of single copy orthologs (Fig. 3A). These strains showed 99.96% identity, attributable to differences in the sequencing strategy adopted and genome coverage (PacBio 100×/454 Roche 33×; Table S2).

We also evaluated the clonal distribution in *N. elongata*. Although we retrieved only 17 genomes to perform this comparative analysis, the topology of the phylogenetic tree using SNPs extracted from the core genome showed a high diversity among *N. elongata* (Fig. 3B). Moreover, we can detect potential phylogroups (e.g., NCTC_10660, M15911, and ATCC_25295) and we emphasize that as soon as new genomes become available, we will be able to further characterize the population structure of *N. elongata*.

*N. elongata* subsp. *nitroreducens* differs from other subspecies because it can reduce nitrate [6]. Subsp. *glycolytica* differs from the others in that it is the only catalase‐positive subspecies [6]. Subsp. *elongata* differs from the others because of its inability to produce acid from d‐glucose [5]. We found seven isolates presenting *katA* gene, including ATCC29315, suggesting that these isolates are catalase‐positive (subsp. *glycolytica*). Another four isolates, including Nel_M001, presented a wide range of genes involved in nitrate reduction (*nar*) (data not shown), suggesting that these isolates belong to subsp. *nitroreducens*. Interestingly, one isolate (1279_NMEN) presented both *katA* and *nar* genes. This indicates that the classification into subspecies criteria defined by Grant (1990) must be reconsidered.

### Resistance and virulence profile

A total of 17 different genes related to antimicrobial resistance were found in all *N. elongata* isolates (Table S3). The observed resistance mechanisms are mainly associated with antibiotic efflux pumps. Eight genes were found in all isolates: *cpxR*, *macAB*, *mtrCDE*, *smeR*, and *tet(35)*. To date, antimicrobial resistance cutoffs for *N. elongata* have not been established. Since its classification as *N. elongata*, the microorganism is described as having sensitivity to several antimicrobials, such as aminoglycosides and aminopenicillins [1, 26].

We observed a very low prevalence of plasmids in *N. elongata*. Only one isolate exhibited IncQ1 plasmid (C2013018262). This isolate was also the only to carry β‐lactamase (*bla*_TEM‐1_) and aminoglycoside‐modifying enzyme genes (*aph(3′)‐Ia*, *aph(3″)‐Ib*, and *aph(6)‐Id*), which are located in the IncQ1 plasmid. The presence of IncQ1 plasmid carrying β‐lactamase resistance genes has been described in *N. gonorrhoeae* and commensal *Neisseria*, such as *N. sicca* [27, 28]. Although plasmids are mainly observed in *N. gonorrhoeae*, commensal species can also harbor plasmids with resistance genes to β‐lactam, sulfonamide, and other antimicrobials [28, 29, 30]. This suggests that *N. elongata*, like other commensal species, can act as a reservoir of antibiotic‐resistance plasmids.

**Fig. 4:**
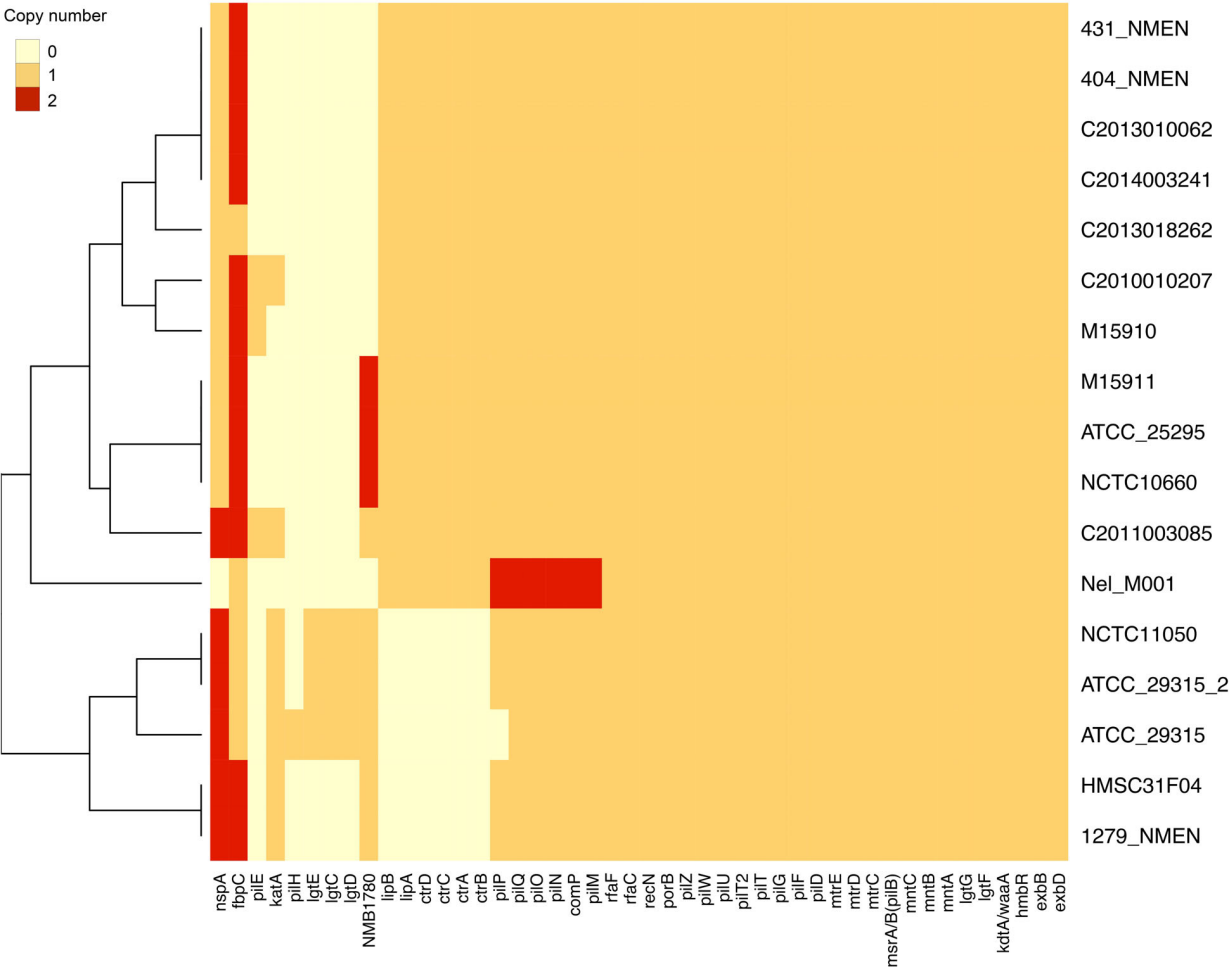
Heatmap of virulence genes detected in *N. elongata* and copy‐number variants. Strains and genes are presented in rows and columns, respectively.

We found 46 genes known to be related to virulence in *Neisseria* (Fig. 4). The isolates NCTC11050, ATCC_29315, ATCC_29315_2, HMSC31F04, and 1279_NMEN were clustered because of the absence of two loci related to capsule formation, *lipAB* and *ctrABCD*. These loci, which encode polysaccharide transporters, were found in most *N. elongata* isolates analyzed in this study, including Nel_M001. Although *N. gonorrhoeae* and some nonpathogenic *Neisseria* spp. have these genes, the lack of the capsule biosynthesis operon *cssABC* restricts this important virulence factor to *N. meningitidis* [3, 31].

A variety of genes responsible for pilus formation (*pil* loci) was detected in all *N. elongata* isolates. There are about 20 types of genes involved in pilus formation, and four are essential for its biogenesis: *pilE*, encoding the major pilus subunit; *pilD*, encoding a peptidase; *pilF*, encoding a protein involved in pilus translocation; and *pilQ*, encoding a subunit that acts as a pore for exit of the pilus [3, 32, 33]. The method adopted in this study to search for virulence genes did not allow identifying *pilE* in some isolates, including Nel_M001 and ATCC29315, because of the low query and hit coverage (data not shown). This can be explained by the virulence database having mostly pathogenic *Neisseria* sequences. Nevertheless, it is known that ATCC29315 carries *pilE* and is able to produce type IV pili [32]. Other nonessential genes for pilus formation were found in virtually all isolates, but only Nel_M001 possessed two copies of *pilP*, *pilQ*, *pilO*, *pilN*, and *pilM*. The presence of more than one copy of these genes needs to be further investigated to understand whether it may improve or not adherence and gene transfer with other *Neisseria* species.

Interestingly, two copies of *nspA* were observed in some isolates and none in Nel_M001. NspA, as well as PorB, is a *Neisseria* surface protein found in *N. meningitidis* and *N. gonorrhoeae* that binds to human factor H, increasing resistance to complement‐mediated killing [34, 35]. The absence of NspA in Nel_M001 could indicate reduced protection against complement‐mediated killing under normal conditions. Nevertheless, the role of NspA as a factor H‐binding protein in *N. elongata* needs to be investigated.

Our results revealed a variety of resistance genes, mostly related to antibiotic efflux pumps, and a low prevalence of plasmids. Although considered antibiotic‐susceptible, *N. elongata* shares some of the same virulence and resistance genes found in other *Neisseria* species. The wide range of virulence genes observed, mostly pilus formation genes, demonstrates that *N. elongata* can adhere and transfer genes to other species. This microorganism has the ability to form vegetations and sometimes abscesses, such as the one occurring in the present case (Nel_M001), making treatment difficult. Therefore, more attention should be given by the medical team to complications of *N. elongata* ‐associated IE. Because these microorganisms are difficult to identify, case reporting and pangenome analyses are crucial for enhancing the understanding of IE pathogenesis and alerting physicians and microbiologists about the need for rapid identification and treatment to avoid unfavorable outcomes. From a genomic perspective, our study provides insights into why this commensal microorganism can cause IE and may assist further biological investigations on nonpathogenic *Neisseria* spp.

## Materials and methods

### Ethical approval

The Institutional Ethics Review Board at the Federal University of Santa Catarina approved this study (CAAE protocol no. 13727619.4.0000.0121) and the patient’s responsible relative signed the informed consent form. The study methodologies were performed according to the standards set by the Declaration of Helsinki.

### Clinical data and literature review

Patient clinical data were retrospectively retrieved from medical records. The case reports articles published up to June 2019 were retrieved from PubMed using *N. elongata* AND IE terms.

### Identification and genome sequencing of *N. elongata* subsp. *nitroreducens* Nel_M001

A Gram‐negative rod‐shaped bacterium was isolated from the blood culture of a patient who presented at the University Hospital of the Federal University of Santa Catarina (Southern Brazil) with intermittent fever for 8 days. The isolate, named Nel_M001, was first identified by the BD BBL™ Crystal™ identification system (Becton, Dickinson, and Company, Franklin Lakes, NJ, USA) as *N. animaloris*. Because this species is uncommon, we decided to perform a new identification using VITEK^®^ MS (BioMérieux S.A., Marcy l’Etoile, France), which revealed the species to be *N. elongata*. Biochemical tests (d‐glucose, catalase, and nitrate reduction) were used to discriminate the subspecies, identifying the isolate as *N. elongata* subsp. *nitroreducens* [6]. Nel_M001 was sent to Neoprospecta Microbiome Technologies (Florianópolis, Brazil) for whole‐genome sequencing. After DNA extraction, the Nextera XT DNA Library Preparation Kit (Illumina Inc., San Diego, CA, USA) was used to prepare a sequencing library. Sequencing was performed by using a MiSeq instrument (Illumina Inc.; paired‐end, 2 × 250 bp) and MiSeq Reagent Kit v2 (Illumina, Inc.).

### Isolates, genome assembly, and annotation

Nel_M001 genome was used in Mash version 2.1.1 [36] to retrieve *N. elongata* isolates with a pairwise distance ≤0.05 from the 2,830 *Neisseria* sp. genomes available in the NCBI database (updated June 2019). A total of 16 N.* elongata* genomes were retrieved, and raw sequencing reads were obtained (Table S2). Read quality was assessed using FastQC version 0.11.5 (https://www.bioinformatics.babraham.ac.uk/projects/fastqc/). Adapters and low‐quality reads were removed using Trimmomatic version 0.35 [37]. Genomes were *de novo* assembled using spades version 3.13.1 [38], and scaffolds were obtained using Gfinisher version 1.4 [39]. Sequences shorter than 500 nucleotides were excluded. The level of completeness was evaluated using BUSCO version 3.0 [40] with beta‐proteobacteria as reference organisms. PlasmidSPAdes version 3.13.1 [41] was used to assemble, and the Plasmid Database (PLSDB) database (updated June 2019) [42] was used to predict plasmid sequences. The genome was annotated with the NCBI Prokaryotic Genome Annotation Pipeline (PGAP) [43]. ANI was assessed using pyani version 0.2.0 using the ANIm method [44].

### Pangenome and phylogenetic analysis

The pangenome of *N. elongata* was inferred with Roary version 3.6.0 [45] using 95% protein clustering. Pangenome openness was estimated with micropan [46] with 1,000 permutations. Two phylogenetic trees were constructed: one based on single copy orthologs and the other based on Core Genome Multilocus Sequence Typing (cgMLST) profiles. Orthologs were determined using UCLUST version 1.2.22q [47] with an identity threshold of 50%. RAxML version 8.2 [48] was used for phylogenetic reconstruction with maximum likelihood, 1,000 bootstrap replicates, and the *N. gonorrhoeae* FA1090 reference genome as the outgroup. Core genes were aligned with MAFFT version 7.271 [49]. Single nucleotide polymorphisms (SNPs) were extracted from core genes using SNP‐sites version 2.3.3 [50]. The SNP alignment was used as input to RAxML version 8.2 [48] to reconstruct the phylogenetic tree using the general time‐reversible model and gamma correction. Since we used only variable sites as input, we used ASC_GTRGAMMA to correct ascertainment bias with the Paul Lewis correction. We used 1000 bootstrap replicates to retrieve the maximum likelihood phylogenetic tree.

### Antimicrobial resistance and virulence analysis

The presence of resistance genes was analyzed using Comprehensive Antibiotic Resistance Database (CARD) version 3.0.3 [51]. Virulence factors were searched against the Virulence Factor Database (VFDB; updated July 2019) [52]. This analysis was conducted for virulence‐associated proteins described for the *Neisseria* genus [47]. UCLUST version 1.2.22q was used to obtain a nonredundant database. The threshold parameters for BLASTp were the same as those applied for resistance gene analysis. All predicted proteins were aligned against these databases using BLASTp with an E‐value cutoff of ≤ 10^−5^, > 60% similarity, and > 50% query and hit coverage.

## Conflict of interest

The authors declare that there are no conflicts of interest.

## Author contributions

MAS, MCS, and MLB designed the study. MAS and HPA conducted the bioinformatic analysis and interpreted the results. MAS collected the clinical data and wrote the first draft of the manuscript. MCS performed the sample isolation and identification. JKP assisted in the bioinformatic analysis. HMM, JMM, and FHB contributed with the laboratory experiments. MLB supervised the study. All authors provided key edits, commented, and approved the final version of the manuscript.

## Supporting information

**Fig. S1.** Echocardiography images from patient. (A) Transthoracic echocardiography suggestive of a 10 mm long pedicled and mobile vegetation on the ventricular face of the aortic metallic prosthesis. (B) Transesophageal echocardiography suggestive of a developing abscess at the base of the aortic prosthesis. (C) Transesophageal echocardiography showing a 2.4 × 1.0 cm abscess at the root of the aorta.

**Fig. S2.** Ranked ANI distribution from *N. elongata* Nel_M001 across *Neisseria* species. Red dotted line represents the threshold used to define species based on ANI. Only the top 50 ranked genomes are shown.

**Table S1.** Summary of case reports of IE caused by *N. elongata*.
**Table S2.** List of *N. elongata* isolates included in the study, and additional data retrieved from GenBank.

**Table S3.** Presence and absence of resistance genes predicted by the Comprehensive Antimicrobial Resistance Database.

## Data Availability

The nucleotide sequence data that support the findings in this study are openly available in the GenBank at NCBI at https://www.ncbi.nlm.nih.gov/nuccore/JAGJWT000000000, accession number JAGJWT000000000.
