# Supplementary figures and images for: Genomic analysis of Neisseria elongata isolate from a patient with infective endocarditis
Source: FEBS Open Bio. 2021 Jun 15;11(7):1987–96. doi: 10.1002/2211-5463.13201 (PMC8406478; doi:10.1002/2211-5463.13201)

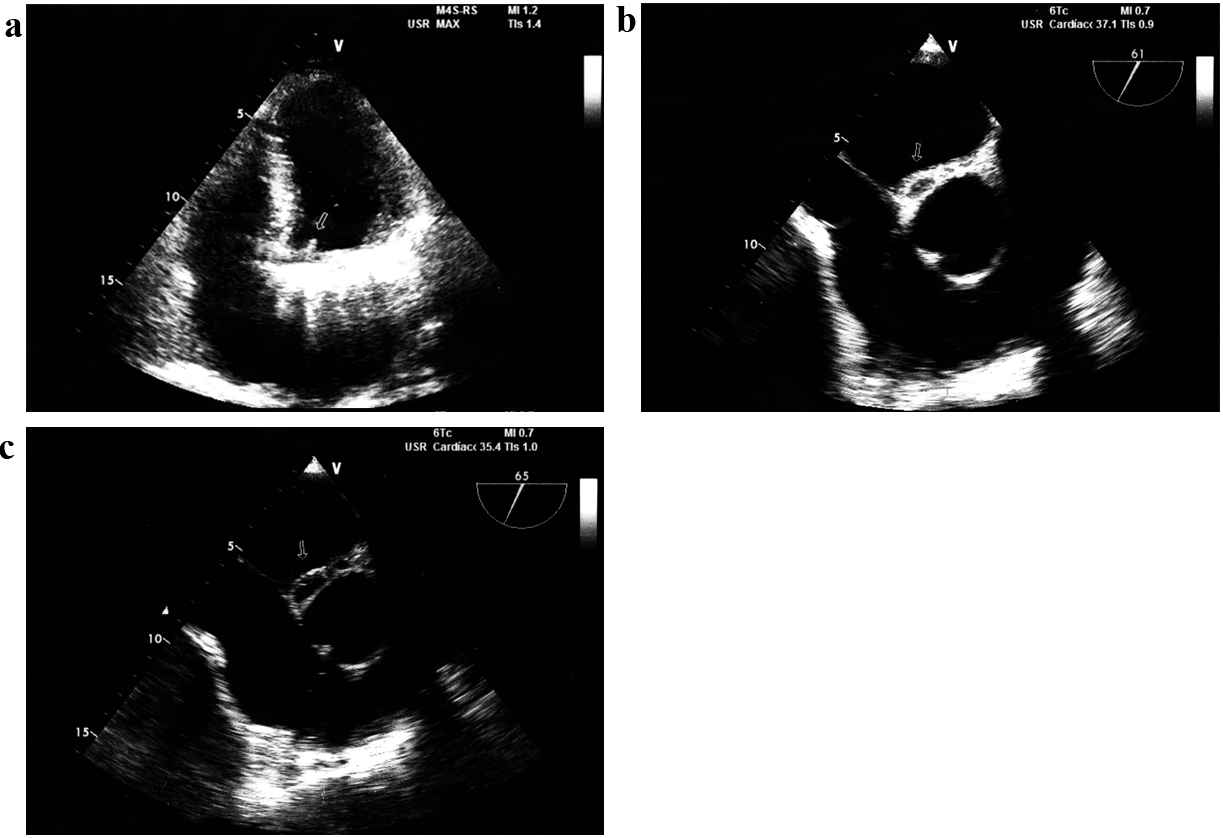

Supplement: Supplementary file 1 — Fig. S1. Echocardiography images from patient. (A) Transthoracic echocardiography suggestive of a 10 mm long pedicled and mobile vegetation on the ventricular face of the aortic metallic prosthesis. (B) Transesophageal echocardiography suggestive of a developing abscess at the base of the aortic prosthesis. (C) Transesophageal echocardiography showing a 2.4 × 1.0 cm abscess at the root of the aorta. [file FEB4-11-1987-s002.tiff]

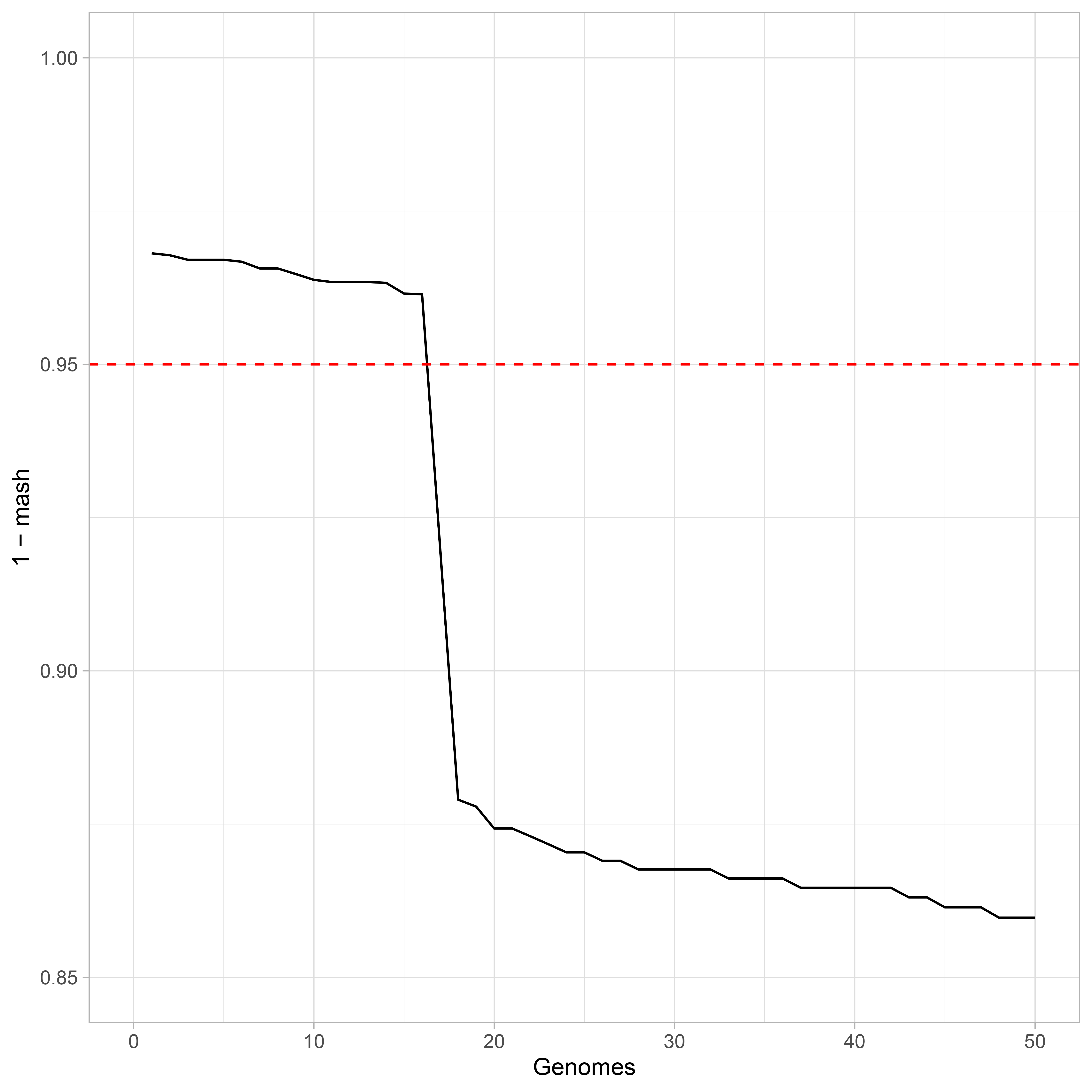

Supplement: Supplementary file 2 — Fig. S2. Ranked ANI distribution from N. elongata Nel_M001 across Neisseria species. Red dotted line represents the threshold used to define species based on ANI. Only the top 50 ranked genomes are shown. [file FEB4-11-1987-s004.tiff]
