# Supplementary material for: Genomic analysis of Neisseria elongata isolate from a patient with infective endocarditis
Source: FEBS Open Bio. 2021 Jun 15;11(7):1987–96. doi: 10.1002/2211-5463.13201 (PMC8406478; doi:10.1002/2211-5463.13201)
Supplement: Supplementary file 3 — Table S1. Summary of case reports of IE caused by N. elongata. Table S2. List of N. elongata isolates included in the study, and additional data retrieved from GenBank. [file FEB4-11-1987-s003.doc]

SUPPLEMENTARY INFORMATION

**Table S1. Summary of case reports of infective endocarditis caused by *Neisseria elongata*.**

| **Reference** | **Patient age (years)** | **Risk factors** | **No. of PBC** | **Subspecies** | **Echo findings** | **Duke criteria** | **Medication/duration** | **Complications** | **Surgery** | **Outcome** |
| --- | --- | --- | --- | --- | --- | --- | --- | --- | --- | --- |
| [2] | 31 | Dental procedure, MVP | 6/6 | *nitroreducens* | MV Veg | 2 major | Penicillin/2 d  Gentamicin/2 d  Ampicillin/4 w | Myocardial abscess, renal failure, CHF | MVR | Hospital discharge |
| [58] | 57 | RHD, AR, infective endocarditis catheterization | 11/11 | *nitroreducens* | None | 1 major, 2 minor^b^ | Ampicillin/43 d Tobramycin/26 d | CHF | AVR | Hospital discharge |
| [59] | 31 | MVP | NS | *nitroreducens* | MV Veg | 1 major, 3 minor | Nafcillin, gentamicin, penicillin/65 d | Stroke | Debridement | Hospital transfer |
| [59] | 25 | None | 6/6 | *nitroreducens* | MR | 2 major | Nafcillin, gentamicin, penicillin/NS | CHF, renal failure | MVR | Hospital discharge |
| [34] | 65 | None | NS | *nitroreducens* | AR, MR | 3 minor^b^ | Ceftriaxone/4 w | TTP, confusion, hemiparesis | None | Hospital discharge |
| [60] | 82 | Dental procedure, myxomatous MV | 4/5 | *nitroreducens* | MV Veg, MR | 2 major | Ceftazidime/10 d Gentamicin/16 d Ampicillin/4 w | CHF, renal failure | MVR | Hospital discharge |
| [36] | 33 | Dental procedure, bicuspid AV | 3/3 | *nitroreducens* | AV Veg, AR, abscess | 2 major | Penicillin/9 d  Ampicillin/4 w Gentamicin/4 w | Myocardial abscess, systemic embolism | AVR | Hospital discharge |
| [61] | 27 | MVP, dental infection | NS | *nitroreducens* | MVP | 1 major, 3 minor | Ceftriaxone/6 w Gentamicin/3 w | Splenic infarction, CHF | MVR | Hospital discharge |
| [28] | 57 | None | 6/6 | *glycolytica* | AV Veg | 2 major | Penicillin/5 w  Gentamicin/5 w | None | None | Hospital discharge |
| [31] | 31 | None | 2/2 | *nitroreducens* | MV Veg | 1 major, 2 minor^b^ | Ampicillin, gentamicin, ceftriaxone/4 w | None | None | Hospital discharge |
| [62] | 74 | Dental infection, bioprosthetic AV | 19/24 | *nitroreducens* | None | 1 major, 2 minor^b^ | Ampicillin/6 w Gentamicin/2 w | None | None | Hospital discharge |
| [32] | 29 | None | 3/5 | *elongata* | MV Veg, MR | 2 major | Ampicillin/3 d  Vancomycin/3 d Ceftriaxone/6 w Gentamicin/6 w | Brachial pseudoaneurysm | Removal of pseudoaneurysm | Hospital discharge |
| [63] | 50 | Prosthetic valve | NS | *nitroreducens* | AV Veg, subvalvular abscess | 1 major, 4 minor | Vancomycin, gentamicin, ampicillin/4 w | Stroke, SAH, subvalvular abscess | AVR | Hospital discharge |
| [64] | 30 | HoCM | 3/3 | *nitroreducens* | None | 1 major, 2 minor^b^ | Benzylpenicillin/2 w Netilmicin/2 w Ceftriaxone/2 w | None | None | Hospital discharge |
| [29] | 65 | None | 2/2 | *elongata* | MV Veg, MR | 1 major, 2 minor^b^ | Ceftriaxone/2 w | None | None | Hospital discharge |
| [35] | 54 | Bicuspid AV, dental procedure | 3/3 | *nitroreducens* | AV Veg, abscess, AR | 2 major | Ceftazidime/7 w Gentamicin/4 w | CHF, aortic root abscess, myocardial abscess | AVR | Hospital discharge |
| [65] | 79 | Prosthetic AV, infected teeth | 3/3 | *nitroreducens* | None | 1 major, 3 minor | Ceftriaxone/6 w Gentamicin/2 w | Azotemia | None | Hospital discharge |
| [66] | 7 | Transposition of the great artery status post Rastelli repair, dental procedure | 4/4 | *nitroreducens* | None | 1 major, 3 minor | Ampicillin/2 w Gentamicin/2 w Ceftriaxone/6 w | None | None | Hospital discharge |
| [26] | 70 | Prosthetic AV,  Klinefelter syndrome | 3/3 | *elongata* | Thickened AVR | NS | Ceftazidime, Gentamicin/NS  Amoxicillin, Gentamicin/3 w  Ceftriaxone/6 w | Renal failure, metabolic acidosis, atrial fibrillation | None | Hospital discharge |
| [8] | 42 | Dental procedure | 2/7 | *glycolytica*^a^ | MV Veg, MR | 1 major, 3 minor | Penicillin/1 w  Gentamicin/1 w Ceftriaxone/7 w | Thalamic infarct, splenic infarct, thalamic brain abscess | MVR | Hospital discharge |
| [30] | 43 | None | 2/2 | *nitroreducens* | MV Veg | 2 major | Ciprofloxacin/7 w | None | None | Hospital discharge |
| [67] | 27 | Cellulitis | 3/3 | *nitroreducens* | TV Veg, VSD | 2 major | Cefazolin/1 d  Vancomycin/2 w | None | None | Hospital discharge |
| [33] | 78 | None | 2/3 | *nitroreducens* | AV Veg, AR | 1 major, 3 minor | Meropenem/4 d Ceftriaxone/2 w Ampicillin/2 w | None | AVR | Hospital discharge |
| [9] | 56 | Recent travel | 2/2 | *nitroreducens* | AV Veg, AR | 2 major, 1 minor | Ceftriaxone/6 w | CHF | AVR | Hospital discharge |
| [68] | 40 | Marfan syndrome | NS | *nitroreducens* | MVP | 1 major, 3 minor | Amoxicillin + clavulanic acid/3 d  Gentamicin/2 d Ceftazidime/3 d | None | None | Hospital discharge |
| This case (2018) | 71 | Dental procedure, prosthetic AV and MV | 2/2 | *nitroreducens* | AV Veg | 1 major, 3 minor | Ceftriaxone, gentamicin/11 d | Aortic root abscess | None | Death |

AR, aortic regurgitation; AV, aortic valve; AVR, aortic valve replacement; CHF, congestive heart failure; HoCM, hypertrophic obstructive cardiomyopathy; MV, mitral valve; MVP, mitral valve prolapse; MVR, mitral valve replacement; MR, mitral regurgitation; NS, not specified; PBC, positive blood cultures; RHD, rheumatic heart disease; SAH, subarachnoid hemorrhage; TTP, thrombotic thrombocytopenic purpura; TV, tricuspid valve; Veg, vegetation; VSD, ventricular septal defect; w, weeks; d, days. ^a^ Based on analysis of 500 bp of the 16S rRNA subunit. ^b^ Possible infective endocarditis according to the modified Duke criteria.

**Table S2.** List of *Neisseria elongata* isolates included in the study, and additional data retrieved from GenBank.

| **Organism** | **Strain** | **BioSample** | **Collection date** | **Geographic location** | **Isolation source** | **Host** | **Seq instrument** | **Gen cov** | **Seq Date** |
| --- | --- | --- | --- | --- | --- | --- | --- | --- | --- |
| *N.* *elongata* subsp. *glycolytica* ATCC 29315 | ATCC 29315_2 | SAMN02797820 | 1970 | USA | Throat | *Homo sapiens* | PacBio | 100× | 14/01/2015 |
| *N.* *elongata* | M15911 | SAMN09704973 | NR | NR | NR | *Homo sapiens* | PacBio | 95× | 01/08/2018 |
| *N.* *elongata* | M15910 | SAMN09704972 | NR | NR | NR | *Homo sapiens* | PacBio | 63× | 01/08/2018 |
| *N.* *elongata* subsp. *glycolytica* | NCTC11050 | SAMEA53328418 | 1970/1976 | Norway, Oslo | Throat swab | *Homo sapiens* | PacBio | 100× | 17/06/2018 |
| *N.* *elongata* subsp. *glycolytica* ATCC 29315 | ATCC 29315 | SAMN00008838 | 1970/1976 | Norway, Oslo | Throat swab | *Homo sapiens* | 454 Roche | 33× | 09/11/2009 |
| *N.* *elongata* subsp. *glycolytica* | 1279_NMEN | SAMN03197249 | unknown | USA, Washington | NR | *Homo sapiens* | Illumina HiSeq | 18× | 10/07/2015 |
| *N.* *elongata* | C2010010207 | SAMN08299212 | 01/01/2010 | USA, Minnesota | NR | *Homo sapiens* | Illumina MiSeq | 18× | 09/04/2018 |
| *N.* *elongata* | C2011003085 | SAMN08299214 | 01/01/2011 | USA, Minnesota | NR | *Homo sapiens* | Illumina MiSeq | 32× | 09/04/2018 |
| *N.* *elongata* | C2013018262 | SAMN08299226 | 01/01/2013 | USA, Minnesota | NR | *Homo sapiens* | Illumina MiSeq | 10× | 09/04/2018 |
| *N.* *elongata* | C2014003241 | SAMN08299229 | 01/01/2014 | USA, Minnesota | NR | *Homo sapiens* | Illumina MiSeq | 20× | 09/04/2018 |
| *N.* *elongata* | C2013010062 | SAMN08299223 | 01/01/2013 | USA, Minnesota | NR | *Homo sapiens* | Illumina MiSeq | 35× | 09/04/2018 |
| *N.* *elongata* subsp. *elongata* | NCTC10660 | SAMEA3672883 | 1900/1969 | NR | Pharynx | *Homo sapiens* | PacBio | 100× | 01/08/2018 |
| *N.* *elongata* subsp. *glycolytica* | 404_NMEN | SAMN03197594 | unknown | USA, WA | NR | *Homo sapiens* | Illumina HiSeq | 25× | 10/07/2015 |
| *N.* *elongata* subsp. *glycolytica* | 431_NMEN | SAMN03197625 | unknown | USA, WA | NR | *Homo sapiens* | Illumina HiSeq | 12× | 10/07/2015 |
| *N.* *elongata* subsp. *elongata* | ATCC 25295 | SAMN05421815 | NR | Norway | Nasopharynx | *Homo sapiens* | Illumina HiSeq | 628× | 02/11/2016 |
| *Neisseria* sp. | HMSC31F04 | SAMN03436258 | NR | NR | Respiratory | *Homo sapiens* | Illumina HiSeq | 220× | 21/10/2016 |
| *N. elongata* subsp. *nitroreducens* | Nel_M001 | SAMN16979883 | 02/07/2018 | Brazil, Florianopolis | Blood culture | *Homo sapiens* | Illumina Miseq | 120× | 13/07/2018 |

Seq, sequencing; Gen cov, genome coverage; NR, not reported.
