# Supplementary material for: Genomic analysis of Neisseria elongata isolate from a patient with infective endocarditis
Source: FEBS Open Bio. 2021 Jun 15;11(7):1987–96. doi: 10.1002/2211-5463.13201 (PMC8406478; doi:10.1002/2211-5463.13201)
Supplement: Supplementary file 4 — Table S3. Presence and absence of resistance genes predicted by the Comprehensive Antimicrobial Resistance Database. [file FEB4-11-1987-s001.docx]

**SUPPORTING INFORMATION**

**Table S3.** **Presence and absence of resistance genes predicted by the Comprehensive Antibiotic Resistance Database.**

| Isolate | Predicted resistance genes | | | | | | | | | | | | | | | | |  |
| --- | --- | --- | --- | --- | --- | --- | --- | --- | --- | --- | --- | --- | --- | --- | --- | --- | --- | --- |
|  | *aph(3')-Ia* | *aph(3'')-Ib* | *aph(6)-Id* | *bla*_TEM-1_ | *sul2* | *emrE* | *lsaC* | *qacH* | *mtrR* | *golS* | *dfrA3* | *rpoB* | *cpxR* | *macAB* | *mtrCDE* | *smeR* | *tet(35)* | |
| 1279_NMEN |  |  |  |  |  |  |  |  |  |  |  |  |  |  |  |  |  | |
| 404_NMEN |  |  |  |  |  |  |  |  |  |  |  |  |  |  |  |  |  | |
| 431_NMEN |  |  |  |  |  |  |  |  |  |  |  |  |  |  |  |  |  | |
| ATCC_25295 |  |  |  |  |  |  |  |  |  |  |  |  |  |  |  |  |  | |
| ATCC_29315 |  |  |  |  |  |  |  |  |  |  |  |  |  |  |  |  |  | |
| ATCC_29315_2 |  |  |  |  |  |  |  |  |  |  |  |  |  |  |  |  |  | |
| C2010010207 |  |  |  |  |  |  |  |  |  |  |  |  |  |  |  |  |  | |
| C2011003085 |  |  |  |  |  |  |  |  |  |  |  |  |  |  |  |  |  | |
| C2013010062 |  |  |  |  |  |  |  |  |  |  |  |  |  |  |  |  |  | |
| C2013018262 |  |  |  |  |  |  |  |  |  |  |  |  |  |  |  |  |  | |
| C2014003241 |  |  |  |  |  |  |  |  |  |  |  |  |  |  |  |  |  | |
| HMSC31F04 |  |  |  |  |  |  |  |  |  |  |  |  |  |  |  |  |  | |
| Nel_M001 |  |  |  |  |  |  |  |  |  |  |  |  |  |  |  |  |  | |
| M15910 |  |  |  |  |  |  |  |  |  |  |  |  |  |  |  |  |  | |
| M15911 |  |  |  |  |  |  |  |  |  |  |  |  |  |  |  |  |  | |
| NCTC10660 |  |  |  |  |  |  |  |  |  |  |  |  |  |  |  |  |  | |
| NCTC11050 |  |  |  |  |  |  |  |  |  |  |  |  |  |  |  |  |  | |

Grey boxes indicate presence of resistance gene.
